# Can serum L-lactate, D-lactate, creatine kinase and I-FABP be used as diagnostic markers in critically ill patients suspected for bowel ischemia

**DOI:** 10.1186/1471-2253-14-111

**Published:** 2014-12-02

**Authors:** Peter HJ van der Voort, Berit Westra, Jos PJ Wester, Rob J Bosman, Ilse van Stijn, Inez-Anne Haagen, Ference J Loupatty, Saskia Rijkenberg

**Affiliations:** Department of Intensive Care Medicine, Onze Lieve Vrouwe Gasthuis, P.O. Box 95500, 1090 HM Amsterdam, The Netherlands; Department of Clinical Chemistry, Onze Lieve Vrouwe Gasthuis, Amsterdam, The Netherlands; Department of Clinical Chemistry, Reinier de Graaf Gasthuis, Delft, The Netherlands; TIAS business school of Tilburg University, Tilburg, The Netherlands

**Keywords:** L-lactate, D-lactate, I-FABP, Creatine kinase, LDH, ALAT, Intestinal ischemia, Bowel, Critically ill

## Abstract

**Background:**

The prognostic value of biochemical tests in critically ill patients with multiple organ failure and suspected bowel ischemia is unknown.

**Methods:**

In a prospective observational cohort study intensive care patients were included when the attending intensivist considered intestinal ischemia in the diagnostic workup at any time during intensive care stay. Patients were only included once. When enrolment was ended each patient was classified as ‘proven intestinal ischemia’, ‘ischemia likely’, ‘ischemia unlikely’ or ‘no intestinal ischemia’. Proven intestinal ischemia was defined as the gross disturbance of blood flow in the bowel, regardless of extent and grade. Classification was based on reports from the operating surgeon, pathology department, endoscopy reports and CT-scan. Lactate dehydrogenase (LDH), creatine kinase (CK), alanine aminotransferase (ALAT), L-lactate were available for the attending physician. D-lactate and intestinal fatty acid binding protein (I-FABP) were analysed later in a batch. I-FABP was only measured in patients with proven ischemia or no ischemia.

**Results:**

For 44 of the 120 included patients definite diagnostic studies were available. 23/44 patients (52%) had proven intestinal ischemia as confirmed by surgery, colonoscopy, autopsy and/or histopathological findings. LDH in these patients was 285 U/l (217–785) vs 287 U/l (189–836) in no-ischemia; p = 0.72. CK was 226 U/l in patients with proven ischemia (126–2145) vs 347 U/l (50–1427), p = 0.88. ALAT was 53 U/l (18–300) vs 34 U/l (14–34), p-0,56. D-lactate 0.41 mmol/l (0.11-0.75) vs 0.56 mmol/l (0.27-0.77), p = 0.46. L-lactate 3.5 mmol/l (2.2-8.4) vs 2.6 mmol/l (1.7-3.9), p = 0.09. I-FABP 2872 pg/ml (229–4340) vs 1020 pg/ml (239–5324), p = 0.98. Patient groups proven and likely ischemia together compared to unlikely and no-ischemia together showed significant higher L-lactate (p = 0.001) and higher D-lactate (p = 0.003).

**Conclusions:**

Measurement of LDH, CK, and ALAT did not discriminate critically ill patients with proven intestinal ischemia from those with definite diagnosis no-ischemia. However, L-lactate and D-lactate levels were higher in patients with proven or likely ischemia and need further study just as I-FABP.

## Background

Critically ill patients occasionally develop ischemia in the splanchnic region. The incidence of clinically relevant ischemia is only known in cardiac surgery patients [1, 2]. The aetiology is diverse but usually mesenteric ischemia is categorized as obstructive or non-obstructive ischemia. The obstructive form is caused by a thrombus or embolus in one of the mesenteric arteries or veins. The non-occlusive mesenteric ischemia (NOMI) develops due to low flow, usually as a result of severe shock [3]. An ischemic bowel may also develop after aortic surgery due to either thrombi or low flow state. In all cases, both the small and the large bowel may be affected. Whatever the aetiology, it is difficult to diagnose intestinal ischemia because of the non-specific clinical signs [4]. Biochemical markers are often used in the diagnostic workup. The use of L-lactate is ubiquitous despite its low sensitivity and specificity [5]. D-lactate, the stereoisomer of the human L-lactate, may be a more specific marker [6, 7]. D-lactate is strictly a product of bacterial fermentation in the gastrointestinal tract including *Escherichia coli, Lactobacillus* species, *Klebsiella*, and *Bacteriodes* species [7]. During intestinal ischemia permeability increases due to decreased mucosal integrity and D-lactate may diffuse into the portal circulation [6, 7]. It has been shown that serum D-lactate levels may be an early indicator for the detection of intestinal ischemia [7, 8]. A study by Assadian et al. showed elevated D-lactate levels in patients with histologically proven ischemic colitis after open aortic reconstruction. More recently, intestinal fatty acid binding protein I-FABP, has gained attention. I-FABP is a small (15-kD) protein within the cytoplasm of mature enterocytes located at the tips of the villi of the small bowel [9]. The concentration is undetectable in normal situations [10–13]. Ischemic damage of the villi leads to release of this protein into the circulation. It has therefore been promoted as a promising new serological marker for intestinal ischemia in animals [11, 14, 15] and humans [16–18].

Prospective studies to the diagnostic value of these serum markers in the specific setting of the intensive care are absent. Hence, the present study is performed to test the prognostic value of biochemical tests in critically ill patients with multiple organ failure and suspected bowel ischemia.

## Methods

### Study objective and design

The objective of this prospective observational cohort study is to determine the predictive value of D-lactate, I-FABP and other more common serological markers of ischemia (L-lactate, lactate dehydrogenase (LDH), creatine kinase (CK) and alanine aminotransferase (ALAT)) in intensive care patients suspected to have intestinal ischemia. The results of L-lactate, LDH, CK and ALAT were readily available for the attending clinician. D-lactate and I-FABP were stored and measured later in one batch at the end of the study. D-lactate levels were additionally measured in a group of healthy volunteers to determine reference levels. At the start, this study did not fall under the Dutch research legislation (WMO) because of its observational character, despite the blood sampling. In virtually all patients a blood sample drawn for other measurements could be used for this study, which limited the number of extra blood sampling. An informal consultation of the local ethic and scientific review board (Medical Ethical Committee on research, Onze Lieve Vrouwe Gasthuis, Amsterdam, The Netherlands) was performed confirming that at that time informed consent was not needed. However, the Dutch ethical committee on research (CCMO) changed their policy concerning blood sampling in 2013, after completion of the study.

### Setting

The ICU where the study was performed is a closed format highest-level mixed medical, surgical and cardiac surgery department with 20 beds in a teaching hospital.

### Patients and sample collection

Over a 24-month period intensive care patients were included when the attending intensivist considered intestinal ischemia in the diagnostic workup. This consideration may have risen on admission or at any moment during intensive care stay. Intestinal ischemia was considered when physical examination and observation of bowel function might be congruent with ischemia. Exclusion criteria other than age below 18 were not defined. As soon as the consideration of intestinal ischemia was raised, a single blood sample of 4 ml was taken to determine serological data.

From each patient only the first blood sample was used in the analysis. Repeated samples were excluded.

### Data collection

Baseline data of all admitted patients in the ICU are recorded in a structured and uniform way in the Patient Data Management System (Metavision®, Tel Aviv, Israel). Data from included patients were retrieved from this database including sex, age, the diagnosis at admission, mortality at the ICU and severity of illness. The Acute Physiologic and Chronic Health Evaluation (APACHE) IV and SOFA scores determined illness severity. Validated data from pathological studies, endoscopy, radiological studies and laparotomies were collected from the hospital information system xCare® (McKesson, Nieuwegein, The Netherlands).

### Patient classification

Patients were classified as ‘proven intestinal ischemia’ , ‘ischemia likely’ , ‘ischemia unlikely’ or ‘no intestinal ischemia’. Classification was based on reports from the operating surgeon, pathology department, endoscopy reports and CT-scan. All available study results were independently analysed by 2 researchers (B.W and P.V.) and categorization was made without prior knowledge of serological markers including plasma D-lactate and I-FABP levels. In case of disagreement between the two researchers consensus was reached by reviewing the data together. Proven intestinal ischemia was defined as the gross disturbance of blood flow in the bowel, regardless of extent and grade. Terms used by pathologist or surgeon like ‘necrotic changes’ , ‘ischemic colitis’ , ‘transmural ischemia’ , ‘ischemic changes in the resected tissue’ , were considered decisive.

### Blood samples

Lactate dehydrogenase (LDH), creatine kinase (CK), alanine aminotransferase (ALAT) and L-lactate were analysed according to instructions of the manufacturer (Roche diagnostics systems, Basel, Switzerland).

The remaining blood of the blood samples were stored in −80°C and analysed for D-lactate and I-FABP in one run after closure of the study inclusion.

D-lactate concentration was spectrophotometrically measured in heparin-plasma. To this end, heparinized blood was centrifuged at 3200 rpm for 10 min in a clinical centrifuge. 500 μL of plasma was deproteinized with 50 μL perchloric acid, mixed on a Vortex for 20 s, and kept on ice for 10 min. Next, the denatured protein solution was centrifuged at 3200 rpm for 10 min. To 350 μL of the supernatant 20 μL KOH was added, mixed for 20 s and kept on ice for 10 min. After centrifugation for 10 min the neutralized-protein-free plasma (NPFP) was used for analysis according to Herrera and co-workers [19]. In brief, D-lactate is oxidized to pyruvate by NAD^+^ in the presence of the D-lactate dehydrogenase. Then pyruvate is enzymatically converted by D-alanine aminotransferase (D-ALT) to D-alanine and 2-oxoglutarate. The latter reaction shifts the equilibrium to the formation of NADH. The amount of NADH formed during the reaction is stoichiometric to the amount of D-lactate in the sample, and it is measured by the increase in absorbance at 340 nm.

The assay mixture contained, in a final volume of 1000 μL: 111 mmol/L glycylglycine pH 10.0, 111 mmol/L glutamate, 4.65 mmol/L NAD^+^, 11.6 U/mL D-alanine aminotransferase and 50 μL heparin-plasma. After a preincubation of 30 s, the reaction was started by addition of D-LDH. The production of NADH was followed in time (10s, 100 s and 200 s) on a Shimadzu spectrophotometer at 340 nm using a molar extinction coefficient of 6300 L/mol/cm. A sample blank to correct the non-specific NAD^+^ transformation was processed using the same analytical conditions as for the analysis of D-lactate but without adding the enzyme D-LDH and was subtracted on all samples.

Literature has shown that D-lactate concentrations will decrease with increasing activities of L-LDH. Indeed, an activity of LDH greater than 1500 IU/L will result in a more than 10% deviation. Hence, in plasma of patients with an LDH activity >1500 the LDH was removed. A reagent blank to compensate for the small, but continuous, non-enzymatic transformation of NAD^+^at alkaline pH was performed in every run and was subtracted from the calibrators, QCs and samples.

Intestinal fatty acid binding protein (I-FABP) was measured in plasma using a commercially available enzyme-linked immunosorbent assay (ELISA) (Hycult Biotechnology b.v., Uden, The Netherlands). The wells of the EIA plate were coated with the monospecific polyclonal antibody (10 μg/ml). First, the samples were diluted twice. To this end, 150 μL diluent buffer was added to 150 μL sample. Next, 100 μL of the standard solution and 100 μL of the diluted sample were added to the plate and incubated for 60 min at 20 C. This was done in duplo. After incubation, 100 μL of the conjugate tracer was added and incubated for 60 min. Next 100 μL of the conjugate Streptavidin-peroxidase was added and incubated for another 60 min. The wells were washed three times with washing buffer (10 mL washing buffer with 390 mL aqua mill) after each incubation. Finally, the reaction was started by adding 100 μL of the tetramethylbenzidine (TMB) substrate (x) every 15 sec to each strip and incubated for another 20 min, and stopped by the addition of citric acid (x) every 15 sec to each strip (and mixed between). The absorbance at 450 nm was measured spectrophotometrically. A standard curve is obtained by plotting the absorbance (linear) versus the corresponding concentrations of the human I-FABP standards (log). The human I-FABP concentrations of samples, which are run concurrently with the standards, were determined from the standard curve.

### Statistical analysis

Continuous variables such as CK, LDH, ALAT, I-FABP, D-lactate and L-lactate were expressed as median and interquartile range (IQR) because of their skewed distribution. The comparison of groups was performed with the Mann–Whitney U-test or the Kruskal-Wallis test were appropriate. Data is shown as absolute numbers and percentage (%). Differences in proportions were evaluated using the Fisher exact test for nominal variables. Sensitivity, specificity, positive predictive value and negative predictive value were calculated according to standard methods. ROC analysis with 95% confidence interval (CI) was performed for the best performing marker, L-lactate, with a positive diagnosis of ischemia tested against the L-lactate level. P-values less than 0.05 were considered statistically significant. Analyses were performed using the statistical software SPSS version 18.0 (SPSS Inc, Chicago, Illinois, USA).

## Results

### Patients

**Figure 1:**
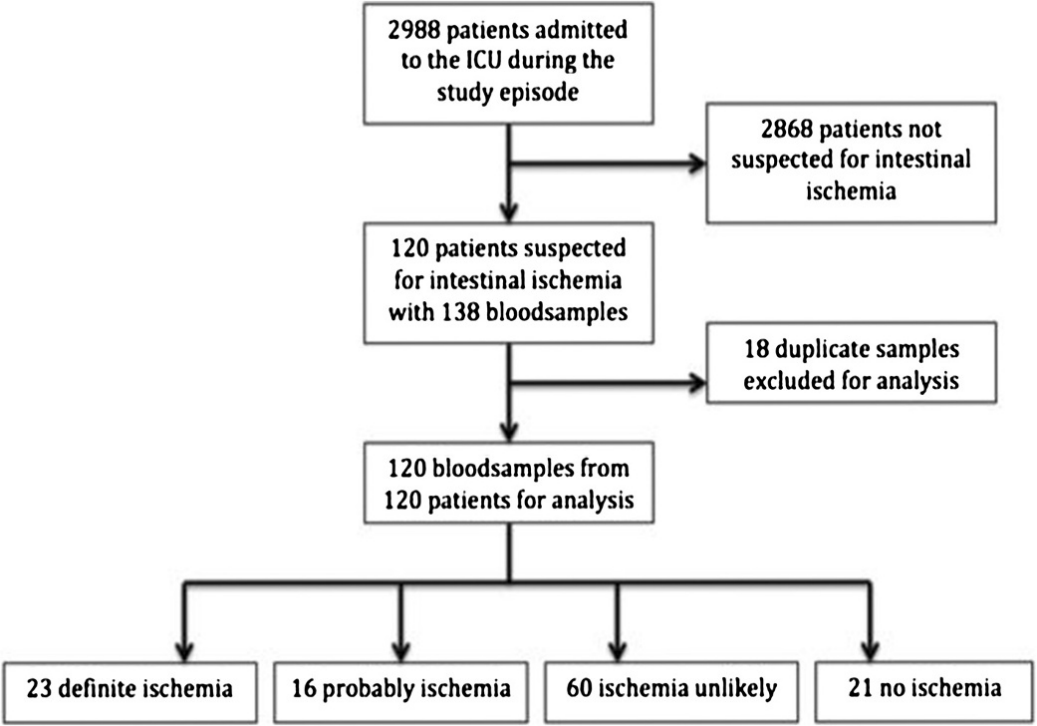
**Flowchart of included patients.**

The study was performed over a period of 24 months. In the study period 2988 patients were admitted to the ICU. 138 samples were collected. 18 samples were duplicate samples of the same patient and therefore excluded for analysis. Baseline characteristics of the 120 included patients are shown in Table 1. The most common diagnosis on admission (39%) in patients with proven intestinal ischemia was other surgery, which included intra-thoracic vascular and heart valve surgery.Figure 1 shows the flow chart for included patients. For 44 of the 120 included patients definite diagnostic studies were available. For the other 76 patients a definite diagnosis could not be made. For the 44 patients with a definite diagnosis, 23 patients (52%) had the diagnosis bowel ischemia confirmed by surgery (n = 20), colonoscopy (n = 2), CT scan (n = 1), autopsy (n = 3) and/or histopathological findings (n = 10). The other 21 patients (48%) underwent surgery (n = 17) and/or autopsy (n = 5) but did not have signs of intestinal ischemia.

**Table 1:** **Baseline characteristics of included patients**

	Proven Intestinal ischemia N = 23	Ischemia likely N = 16	Ischemia unlikely N = 60	No intestinal ischemia N = 21	***P*** -value ^*^
Age (years)	73 (64.0 – 78.0)	67 (63 – 77)	69 (61 – 77)	70.0 (62.0 – 77.5)	0.56
Male	9 (43)	11 (69)	25 (42)	14 (67)	0.08
APACHE IV predicted mortality	0.49 (0,26-0.73)	0.42 (0.13 – 0.79)	0.45 (0.24 – 0.73)	0.45 (0.27 – 0.61)	0.53
Diagnosis on admission (%)					
Severe sepsis & septic shock	5 (21.7)	2 (12.5)	7 (11.7)	9 (42.6)	
Cardiogenic shock	1 (4.3)	1 (6.3)	7 (11.7)	1 (4.8)	
Hypovolemic shock	1 (4.3)	3 (19)	2 (3.3)	-	
After CPR	-	-	12 (20)	1 (4.8)	
Aortic aneurysm repair	3 (13)	5 (31.3)	8 (13.3)	3 (13)	
After other surgery	9 (39)	4 (25)	11 (18.3)	5 (23.8)	
Other	4 (17.4)	1 (6.3)	13 (21.7)	2 (9.5)	
Mortality	13 (57)	4 (25)	15 (25)	7 (33)	0.14

All values are medians and interquartile ranges or absolute number with (%).

^*^p-value of ‘Proven intestinal ischemia’ compared to ‘No intestinal ischemia’.

20 patients with intestinal ischemia underwent laparotomy, which confirmed ischemia and underwent subsequent surgical resection (87%), while 17 patients without intestinal ischemia also had abdominal surgery (76%).

### D-lactate

**Figure 2:**
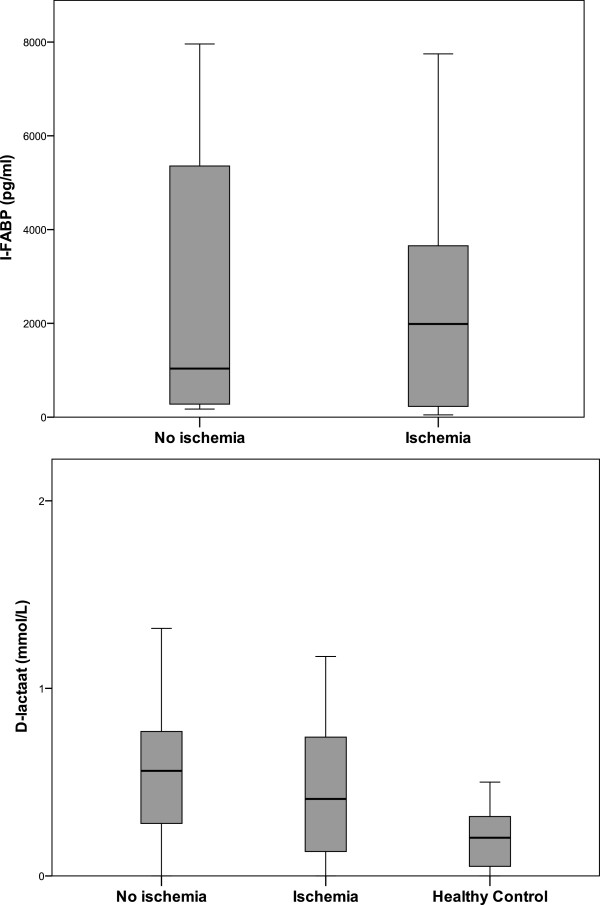
**Boxplots of I-FABP and D-lactate in patients with and without intestinal ischemia.**

D-lactate levels were measured in all patients and in healthy controls. The results are shown in Table 2 and Figure 2. A significant difference between healthy controls and patients with proven or no intestinal ischemia was found (p = 0.023 and p = 0.001, respectively). However, there was no difference between patients with proven compared to no intestinal ischemia (p = 0.46). When all groups were taken into account, no statistical difference was found between groups (p = 0.099). The combined group proven and likely ischemia together compared to unlikely and no-ischemia together showed D-lactate levels of 0.79 (IQR 0.49-1.16) versus 0.65 (IQR 0.37-0.94); p = 0.003.

**Table 2:** **Results of D-lactate, I-FABP, L-lactate and ALAT, CK, LDH levels**

	Proven Intestinal ischemia N = 23	Ischemia likely N = 16	Ischemia unlikely N = 60	No intestinal ischemia N = 21	Healthy control N = 27	p-value ^$^	p-value ^&^
D-lactate (mmol/l)	0.41 (0.11 – 0.75)	0.54 (0.34-0.90)	0.33 (0.08-0.62)	0.56 (0.27 – 0.77)	0.20 (0.038 – 0.33)	0.003	0.46
I-FABP (pg/ml)	2872 (229–4340)	Not available	Not available	1020 (239–5324)	Not available	Not available	0.98
L-lactate (mmol/L)	3.5 (2.2 – 8.4)	4.1 (2.8-6.7)	1.8 (1.2-3.0)^#^	2.6 (1.7 – 3.9)	Not available	0.001	0.09
ALAT (U/I)	53 (18 – 390)	67 (20–637)	79 (33–204)	34 (14 – 34)	Not available	0.77	0.56
CK (U/I)	226 (126 – 2145)	430 (169–992)	631 (161–1699)	347 (50 – 1427)	Not available	0.51	0.88
LDH (U/I)	285 (217 – 785)	366 (250–1586)	435 (282–752)	287 (189 – 836)	Not available	0.52	0.72

All values are medians and interquartile ranges. ^*^p-value represents proven compared to no intestinal ischemia. ^#^p = 0.002 for ischemia likely vs unlikely. ^$^p-value represents proven and likely together compared to unlikely and no ischemia together. &p-value represents proven compared to no ischemia.

### I-FABP

I-FABP was only measured in the groups proven and no intestinal ischemia. Patients with intestinal ischemia (Figure 2) showed a non-significantly higher median I-FABP level compared to those without intestinal ischemia (2872 vs 1020 pg/ml, p = 0.98).

### CK, LDH and ALAT

**Figure 3:**
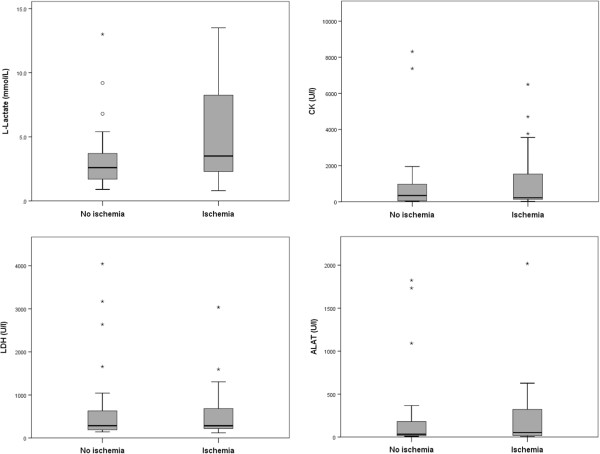
**Boxplots of L-lactate, CK, LDH and ALAT in patients with and without intestinal ischemia.**

ALAT, CK and LDH levels were measured in all patients and data is shown in Table 2 and Figure 3. No significant differences were shown between separate groups. When all groups were taken into account, no statistical difference was found between groups (p = 0.43 for ALAT, p = 0.53 for CK and p = 0.20 for LDH). The combined groups proven and likely ischemia together compared to unlikely and no-ischemia together showed CK levels of 258 (IQR 156–1024) versus 562 (IQR 129–1686); p = 0.51. The combined groups proven and likely ischemia together compared to unlikely and no-ischemia together showed LDH levels of 350 (IQR 225–785) versus 399 (IQR 243–751); p = 0.52. The combined groups proven and likely ischemia together compared to unlikely and no-ischemia together showed ALAT levels of 61 (IQR 18–390) versus 70 (IQR 25–204); p = 0.77.

### L-lactate

**Figure 4:**
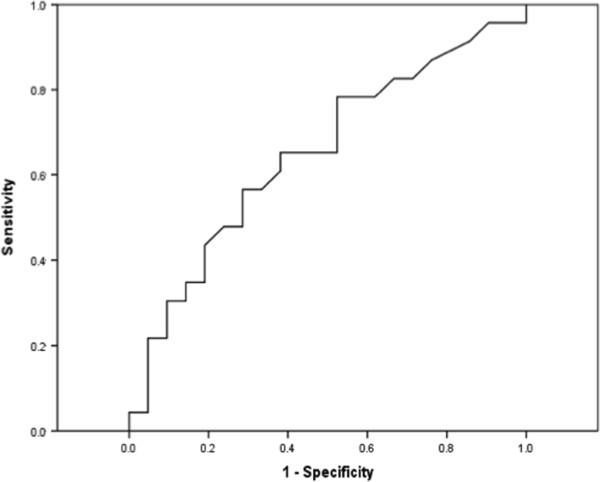
**ROC curve of L-lactate to detect intestinal ischemia.**

When all groups were taken into account, a statistical significant difference was found between groups (p = 0.001). However, between individual groups a statistical significant difference was present only in the comparison between ischemia likely and unlikely (p = 0.002). The median L-lactate levels in patients with proven and no ischemia were 3.5 mmol/L (2.2-8.4) and 2.6 mmol/L (1.7-3.9), respectively (p = 0.09). The combined groups proven and likely together compared to unlikely and no-ischemia together showed L-lactate levels of 3.9 (IQR 2.4-7.4) versus 1.9 (IQR 1.3-3.2); p = 0.001.We determined the best cut-off point to differentiate patients with proven ischemia and no intestinal ischemia by using the sum of maximum sensitivity and specificity. In a ROC-analysis (Figure 4) showed an area under curve of 0.65 (95%CI 0.49-0.81, p = 0.09). Using a cut-off point of 2.2 mmol/L the sensitivity was 78% and the specificity was 48%.

## Discussion

The diagnostic process concerning intestinal ischemic in critically ill patients on the intensive care unit is challenging. The present study of prospectively collected biochemical markers shows that these data would, in retrospect, not have helped the clinician to make the diagnosis more easily when only patients with proven ischemia and no ischemia were taken into account. The most common biochemical marker, L-lactate, was in general higher in patients with compared to patients without a definite diagnosis of intestinal ischemia but it could not discriminate between these patients sufficiently in the clinical setting. Combined group of proven and likely ischemia did show higher L-lactate compared to patients unlikely or no ischemia. Unsuccessful discrimination was the case for creatine kinase, lactate dehydrogenase and ALAT. In addition, we studied D-lactate and I-FABP as they were recently linked to high diagnostic accuracy in patients in the emergency ward and in-hospital [6, 20, 21]. D-lactate is exclusively produced by the indigenous bowel flora. An ischemic mucosa may lead to translocation of D-lactate that can be measured in plasma. Indeed, compared to healthy controls, D-lactate levels were elevated. However, in critically ill patients with and without proven intestinal ischemia serum D-lactate levels were equally elevated but the combined group proven and likely ischemia had a significantly higher D-lactate. I-FABP has recently become available and is specifically released by ischemic mucosa. The range of I-FABP levels was broad in patients with and without definite bowel ischemia. As renal function has not been proven to be a factor determining the level of these biomarkers we did not take this into account.

Clinical symptoms have a low diagnostic power to detect NOMI [4]. In the intensive care setting symptoms like inability of enteral feeding may suggest an ischemic bowel but often occur in the absence of ischemia as well and there is a lack of relevant clinical studies in the critically ill. In additional to clinical symptoms, now the serological markers appear to have a low discriminating power in the diagnostic process of intestinal ischemia as well. This may be explained by several reasons. First, the test characteristics of a diagnostic test are determined by the population were it is tested. Most serological tests that are used in the intensive care have been developed for in-hospital patients in general or specific populations (for instance patients after vascular surgery) and have not been tested in critically ill patients in particular. The present cohort of patients was even a subset of critically ill patients as they were selected for their suspected splanchnic ischemia. The severity of illness, illustrated by the APACHE IV system, is high. In this specific population marginal splanchnic perfusion might be highly prevalent, which is suggested by the high L-lactate and the high I-FABP in all patients. For the same reason, the results of the present study cannot be extrapolated to non-critically ill patients. Second, it is likely that some patients in whom a definite diagnosis could not be made did have intestinal ischemia. The sample size of this study is relatively small and it might be argued that a larger trial is still needed to determine the exact value of the biochemical markers, which may be particularly true for L-lactate, D-lactate and I-FABP.

Our results match with the study from Block et al. [22] although their population was different. They found that in patients who presented with an acute abdomen, lactate and I-FABP did not discriminate between ischemia or no ischemia. The majority of their patients had obstructive mesenteric ischemia where most critically ill patients have NOMI due to low flow. Block et al. found D-dimer discriminating for ischemia [22]. As most of critically ill patients with organ failure and central lines have an increased D-dimer, we did not take that into account. Poeze et al. [6] studied D-lactate in patients after aortic aneurysm repair with and without ischemic complications. D-lactate discriminated adequately between these two groups. We could not confirm this finding as in our subsets of patients with aortic aneurysm repair a significant difference in any of the markers was not present. Assadian et al., in concordance with our study, did not find different D-lactate levels in ischemic patients compared to non-ischemic patients [8]. Collange et al. could not find an elevated D-lactate level perioperative [23]. Thuijls et al. found in a study in emergency room and in-hospital patients with acute abdomen and suspected for intestinal ischemia that plasma I-FABP was significantly higher in patients with ischemia compared to patients without ischemia [20]. In our patient population of critically ill patients this was not the case. It is likely to be explained by the mucosal ischemia, which is highly prevalent in severely ill patients. In addition, we did not measure I-FABP in the likely and unlikely groups. The same group of researchers found for I-FABP a sensitivity of 100% and a specificity of 98% for patients who developed intestinal necrosis after aortic surgery [21]. In our subset of patients we could not confirm this finding. There is some discussion concerning the use of the different I-FABP ELISA in humans but it remains speculative what the results would have been when using a different test [24].

Several limitations of the study need discussion. A ‘golden standard’ for the diagnosis of bowel ischemia is lacking for 76 out of 120 patients. An unknown proportion of these patients will have suffered from an ischemic bowel. However, these patients did not have surgery or autopsy to confirm the diagnosis. As a consequence, bias may have occurred. In addition, some patients may have had temporarily ischemia, which resolved without surgery. There is an interaction between mortality and the categorization of patients. Bowel ischemia is associated with a higher mortality, which may lead to autopsy and autopsy is a major tool to detect proven ischemia. As a result, autopsy has predominantly been performed in patients with definite ischemia or no ischemia. The mortality in both groups ischemia likely or unlikely is lower (Table 2) and, as a consequence, autopsy is less frequently performed. When we combine proven and likely ischemia and compare this new group with a combined group unlikely and no ischemia then L-lactate and D-lactate were significantly different between these two groups (Table 2). Some of these patients probably had temporarily ischemia but did not die because of an ischemic bowel and autopsy did not take place. Above considerations make clear that, unavoidably, the study suffers from some extend of selection as only 44 patients had a definite diagnosis. Nevertheless, this study is the first and largest that determined in a clinical setting the usefulness of biochemical markers in the diagnostic process of intestinal ischemia. We conclude that it is unlikely that biochemical results of, CK, LDH, and ALAT can lead the physician in clinical decision making concerning critically ill patients with suspected intestinal ischemia. For L-lactate, D-lactate and I-FABP more studies are needed.

## Conclusion

It is unlikely that biochemical results of, CK, LDH, and ALAT can lead the physician in clinical decision making concerning critically ill patients with suspected intestinal ischemia. For L-lactate, D-lactate and I-FABP more studies are needed.

### Key messages

- Intestinal ischemia in the critically ill patient is difficult to diagnose
- Biochemical tests are frequently used in the diagnostic workup in patients with suspected intestinal ischemia
- A L-lactate serum level of 2.2 mmol/l shows a sensitivity of 78% and specificity of 48% and a ROC value of 0.65
- LDH, ALAT and CK could not discriminate between patient with and without an ischemic bowel
- The specific markers L-lactate, D-lactate and I-FABP may be useful in case of intestinal ischemia in critically ill patients but more studies are needed.

## Authors’ information

PV and SR have an academic degree in clinical epidemiology.

## Abbreviations

NOMI: Non-occlusive mesenteric ischemia
LDH: Lactate dehydrogenase
ALAT: Alanine transaminase
CK: Creatinekinase
I-FABP: Intestinal fatty acid binding protein
ICU: Intensive care unit
APACHE: Acute physiology and chronic health evaluation
SOFA: Sequential organ failure assessment
KOH: Potassium hydroxide
NPFP: Neutralized-protein-free plasma
NAD+: Aldehyde dehydrogenase
NADH: Nicotinamide adenine dinucleotide
D-ALT: D-alanine aminotransferase
ELISA: Enzyme-linked immunosorbent assay
TMB: Tetramethylbenzidine
IQR: Interquartile range
ROC: Receiver operating characteristics
CI: Confidence interval.
